# Diagnosis between Epididymitis and Orchitis

**Published:** 1843-12

**Authors:** 


*Diagnosis between Epididymitis and Orchitis.*—In the former disease the epidiymis forms a distinct crescentic tumour behind the testis, and is hard, while the testis preserves its proper size and elasticity, and is free from adhesions. In orchitis, on the contrary, the testis is increased in size, hardened, and adherent to the skin of the scrotum, which has lost its puckered appearance.—*Ricord.*

